# Has a New Day Dawned?

**Published:** 1890-12-06

**Authors:** 


### The Doctor's Armchair.

### HAS A NEW DAY DAWNED?

Koch follows hard upon Pasteur: Pasteur succeeded Jenner by a long interval. What is the special significance of these modern departures? Medical men, and even the intelligent portion of the public, ought to be able to see great possibilities in the line of research, prevention, and cure, instituted by Jenner and followed out by Pasteur and Koch. Two main questions seem to comprehend the situation at the present moment. Is it the fact that the larger number of human diseases are caused by the abnormal situation and activity of different bacterial forms? That is the first question. And may we hope so to keep these bacterial forms in check by means of inoculations as to prevent them producing diseases, or to cure the diseases which result from their activity, in their earlier stages?

It will be seen that if most diseases have a bacterial origin a complete revolution of medical methods is imperative and imminent. What is the attitude of the practical physician towards disease in general at the present moment? It is one of watchfulness, of guidance of the patient through dangerous ways, of protection against external sources of harm, of anticipation of possible dangers and injuries in the near future, of, in fact, the piloting of a vessel, that has encountered wars and storms, through dangerous shallows and rocky places into the haven of security and health. Doctors are bred in these principles; their forefathers were bred in them. The average medical man when he is called to a case of diphtheria, typhoid fever, pneumonia, consumption, scarlet fever, ulceration of bone or skin, or any other common disease, does not expect by the application of a plaster, or the administration of a potion, to restore his patient to health in a day. On the contrary, he knows absolutely that each disease will run its course and terminate, either in the death of the patient, or in the successful resistance by nature of its advancing course. The doctor's function, as we have said, is to watch the patient, to guard him against external dangers, and to administer to him some of those manifold remedies which arouse, stimulate, and strengthen natural forces to resist the advance of the disease.

For a moment let us consider how medical men have come by their present attitude of mind. Experience, the experience of at least six thousand years, has been their school dame and their imperious task-mistress. Necessity has driven doctors into the somewhat narrow ways of mere bedside watching and practice. The most clear sighted and honest among them have found out for themselves that there has been nothing else to be done but to watch, to guard, to anticipate, and to try on each new case those methods which experience has proved to be serviceable to many patients and in the hands of many physicians in past times. This is a brief description of the orthodox medical position of the present period, and of its origin.

But now, a new day seems to be dawning. Certain men appear to be penetrating much more deeply into the secrets of nature than formerly. They say that they see the causes at work which produce diseases, with all their symptoms: they say that if they could lay a controlling hand upon the causes, the diseases would no longer harass and destroy mankind: they say that in certain cases they have laid their controlling hands upon the causes; and they announce their confident expectation of detecting, one by one, the remaining enemies which attack man in the citadel of his life, and of rendering them powerless for harm. This is a wonderful expectation. Do the men who hold it see the greatness of its proportions? Does the civilised, progressive, and scientific man of the present time perceive clearly the goal to which he thinks he is tending? If the bacteriologist and the inoculator are right in their judgment as to the results of their past experiments, and in their anticipations as to the victories which await them, we are at the beginning of an era such as the world has never seen, and has never expected to see. The new departure is not a quickened impulse carrying us along old lines; it is a revolution; it is the fruit of a revelation; it will bring about a new humanity; a humanity practically without sickness or disease; a humanity which none have ever expected to see except those who have believed that at dissolution "this mortal shall put on immortality." Now this, as we have said, is a wonderful promise. But experience shows that the doubtfulness of fulfilment is proportionate to the greatness of the promise. If a man promise his neighbour a golden apple, his neighbour will expect to enjoy the fruit at the proper season, because he understands a figure of speech. But if he promise him an apple of gold, the neighbour will do well to indulge no expectations at all. Even if the gold should come he will be wise to take it to the assayers before he sits down to write his letter of thanks.

Are we, then, altogether sceptics in the affairs of science and medicine? We are not sceptics at all, either in things natural or in things spiritual. We believe it would be quite easy for the infinite power that is immanent in, and rules all things, to make human beings quite impervious to disease; to destroy the wondrous organisms which are said to produce disorder; to destroy or to make them as advantageous to man as they now appear to be injurious. We believe that the divine power may so purify the vision and strengthen and enlarge the intellect of man as that he shall see clearly what he now hesitatingly thinks he sees, and shall do with the utmost certainty what he now only hopes he is accomplishing. Of course, we believe all these things, because no man who has risen to the apprehension of an infinite mind can help believing them.

But that is not the question. The question is not—what is possible to God and to man; it is—what has been accomplished? And what, judging from the past history and doings of the human race, is likely to be accomplished? Every man who has any just measure of the value and possibilities of mind must hope that Jenner was right, that Pasteur was right, that Koch is right, and that all those promises of conquest which rise before the scientific vision may be fulfilled a thousand-fold. We are among those who hold that the progress of the human race knows neither check nor limit; that what is incomprehensible here will be clear and luminous to those human spirits which attain to immortality. But for the very reason that these are our faiths and our hopes, we will not accept delusions instead of their realisation. Proofs—strong, clear, convincing, irresistible—these are what we ask for, and insist upon; and nothing else will satisfy either the human conscience or the scientific mind.

In the meantime, and during the period of expectation, what is the daily duty of every medical man to his patients, and to the science of medicine? To each patient he must give such homely, constant, and intelligent help as he has proved by experience to be undoubtedly serviceable. He must not desert the beaten path, until a way is shown to him which has been proved safer and easier than it. No one doubts the possibility of going from London to Edinburgh in a balloon; but most people would prefer to go by the Scotch express, or by old-fashioned coach; or even to be wheeled in a wheelbarrow if they wished to get there within some certain definite time. All of us have proved the old ways; we will stick to them until the new are shown to be better. At the same time no wise man will close either his eyes or his ears, and no sane man will refuse to empty his pockets of bronze, if he can find gold wherewith to fill them. Every loyal soldier in the army of science will march forward with his regiment. All the new worlds that lie before him he will take his share in conquering. Only, if he be a man endowed with the noblest sort of intellect, he will not put off his armour until the battle is ended, and he will not blow the trumpet of victory whilst the enemy in countless numbers and unmeasured strength is still facing him in the field.

